# Exposure to 1.8 GHz radiofrequency field modulates ROS in human HEK293 cells as a function of signal amplitude

**DOI:** 10.1080/19420889.2022.2027698

**Published:** 2022-02-03

**Authors:** Marootpong Pooam, Nathalie Jourdan, Blanche Aguida, Cyril Dahon, Soria Baouz, Colin Terry, Haider Raad, Margaret Ahmad

**Affiliations:** a Department of Biology, Faculty of Science, Naresuan University, Phitsanulok, Thailand; b Sorbonne Université – CNRS, Paris, France; c Department of Biology, Xavier University, Cincinnati, Ohio, USA

**Keywords:** Electromagnetic fields, radiofrequency, reactive oxygen species, hormesis, ros signaling

## Abstract

The modern telecommunications industry is ubiquitous throughout the world, with a significant percentage of the population using cellular phones on a daily basis. The possible physiological consequences of wireless emissions in the GHz range are therefore of major interest, but remain poorly understood. Here, we show that exposure to a 1.8 GHz carrier frequency in the amplitude range of household telecommunications induces the formation of ROS (Reactive Oxygen Species) in human HEK293 cultured cells. The ROS concentrations detected by fluorescent imaging techniques increased significantly after 15 minutes of RF field exposure, and were localized to both nuclear and cytosolic cellular compartments. qPCR analysis showed altered gene expression of both anti-oxidative (SOD, GPX, GPX, and CAT) and oxidative (Nox-2) enzymes. In addition, multiple genes previously identified as responsive to static magnetic fields were found to also be regulated by RF, suggesting common features in response mechanisms. By contrast, many RF effects showed evidence of hormesis, whereby biological responsivity does not occur linearly as a function of signal amplitude. Instead, biphasic dose response curves occur with ‘blind’ spots at certain signal amplitudes where no measureable response occurs. We conclude that modulation of intracellular ROS can be a direct consequence of RF exposure dependent on signal frequency and amplitude. Since changes in intracellular ROS may have both harmful and beneficial effects, these could provide the basis for many reported physiological effects of RF exposure.

## Introduction

Man-made oscillating electromagnetic fields are ubiquitous in the modern world and include a relatively low-frequency ELF-MF of 10–300 Hz (e.g. from electric power grids and biomedical pulsed field devices) as well as radio frequencies in the MHz range (from electrical appliances, television, and radio) and in the GHz range emanating from modern telecommunications and microwave ovens [1,2].

Because they are so widespread and in such constant use, the possible physiological effects of non-thermal emanations from cellular phones and Wi-Fi on the human body have generated concern [3–5] and given rise to a large literature including on the physiological consequences in the brain and nervous system; in the reproductive system including sperm formation; in the onset of cancers; and in many other health-related conditions (for recent reviews see e.g. [6–9]). However, given the wide range of experimental protocols and signal parameters used in the different studies, as well as frequently contradictory results, many of these effects remain controversial and poorly resolved.

Despite this overall lack of clarity, a recurring theme in the literature has been that free radicals and ROS (reactive oxygen species) are induced in living cells by exposure to non-thermal radiofrequency fields [10–12]. These observations are important for a number of reasons. First, ROS (reactive oxygen species) provide a chemical means whereby a single primary response mechanism (manipulating cellular levels of ROS) can mediate a multiplicity of biological effects. In essence, ROS are chemically highly reactive oxygen species [13] and include peroxides, superoxides, hydroxyl radicals, and singlet oxygen. ROS are generally formed as a byproduct of the normal metabolism of oxygen by mitochondrial, chloroplast, and cell membrane associated enzymes, but are also induced by environmental stress (e.g., pollution, UV, or heat exposure).

If produced in excess, ROS can cause significant damage to cell structures and DNA as a consequence of irreversible nucleic acid, lipid, and protein oxidation [13–15]. On the other hand, at lower concentration, ROS can have milder effects on cellular processes, regulate many cellular signaling pathways, and even have beneficial or therapeutic effects [15,16]. As a consequence, many of the disparate biological processes reported occurring as a result of radiofrequency exposure, including both harmful and beneficial effects, could in principle be explained by a primary effect of the radiofrequency field on modulating the concentration of cellular ROS.

A second reason why ROS modulation presents a promising hypothesis to explain radiofrequency effects is that there is considerable precedent for biological systems to respond in this way to a range of electromagnetic fields. In particular, both static magnetic fields and low frequency pulsed electromagnetic fields (PEMFs) have been reported to modulate ROS and ROS signaling pathways in a large range of organisms [16–23]. Exploring these mechanisms for possible common features may be a promising avenue to elucidate underlying mechanisms and cellular targets.

In the present study, we have exposed cells to a simplified experimental protocol. The signal is a basic single-frequency sinusoidal wave (1.8 GHz) in an amplitude range of between −8.5 and −76 dBm. Only a single, short exposure time (15 min) was applied, and analysis was conducted no later than 3 hours after exposure. In this way, we sought to detect physiological effects related to primary cellular receptor responses rather than pursue indirect effects unrelated to the physiological target.

## Materials and Methods

### 1. Cell cultures and growth conditions

Cell cultures and growth conditions were as previously described [21]. Briefly, human embryonic kidney (HEK) 293 cells were cultured in a CO_2_ incubator (MCO-18AC, Panasonic Biomedical, Leicestershire, UK), at 37°C and 5% CO_2_. Cells were grown in 75 ml culture flasks containing 10 ml Modified Eagle medium (MEM; Sigma, Sigma, St Louis, MO) and sub-cultured every 4 days. The static magnetic field inside the incubator was at 40 microT. Sham and test samples were diluted from the same parent cell culture stock and placed at identical positions in the incubator for test and mock exposure.

### 2. Oscillating Electromagnetic Field Exposure Conditions

Cell cultures were exposed to an RF signal generated by a 1.8 GHz transmitting antenna, which is connected to an IFR 2026 10 kHz to 2.4 GHz multisource signal generator.

The design and analysis of the 1.8 GHz printed monopole antenna used in the experimental setup have been carried out using the full wave simulation package CST Microwave Studio, which is based on the Finite Integration Technique (FIT).

**Figure 1.**
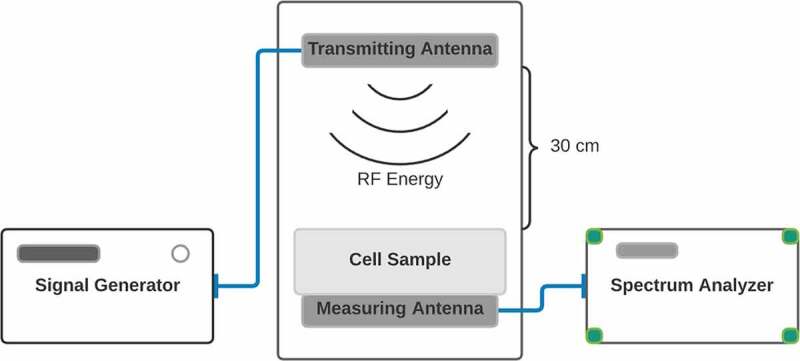
**Diagram of Experimental Setup**. Top: Image of the transmitting antenna. The structure is printed on a Kapton Polyimide substrate. The final optimized design is fabricated by inkjetting a conductive ink based on silver nano-particles by a material printer. Bottom: Placement of antennae in incubator. The transmitting antenna was positioned at 30 centimeters above the sample within the incubator (central square panel). The measuring antenna was placed at the sample position for measurement before and after the experiment. The spectrum analyzer and signal generator were located outside of the incubator.

As shown in Figure 1, the antenna consists of a square winding radiating element with a central gap and fed by a coplanar waveguide. The antenna structure is printed on a Kapton Polyimide substrate with a dielectric constant of 3.4 and a loss tangent of 0.002. The final optimized design is fabricated by inkjetting a conductive ink based on silver nano-particles by a Fuji-Dimatrix material printer. The antenna's return loss was measured using an Agilent PNA-X series N5242A Vector Network Analyzer (VNA) with (10 MHz–26.5 GHz) frequency range and it was found to be resonating at 1.8 GHz with a return loss of 24 dB.

The transmitting antenna was positioned at 30 cm above the sample. Intensity of the signal was measured before and after the experiment using an ANRITSU MS2711D Spectrum analyzer at the position of the sample. Cells were exposed to RF intensities in a monolayer for 15 minute intervals as follows: Control (background radiation): −96 dBm; High: −8.5 dBm; Medium: – 31 dBm; Low: – 55 dBm; Very Low: – 67 dBm. The control condition (no signal) exposure was performed by placing cells under the antenna for fifteen minutes but without exposure (antennae off). Five duplicate experiments were performed for each experimental conditions, on different days and using different prepared cell cultures. Sample temperature was unchanged during exposure.

### 3. Intracellular localization of ROS in human HEK293 cell cultures using confocal imaging techniques

Living human HEK cells seeded in cell observation chambers were incubated in 40 mM potassium phosphate buffer (pH 7) containing 12,5 M DCFH-DA (Molecular Probes) for 15 min in a 95% air–5% CO_2_ incubator at 37°C and were simultaneously exposed or not to RF for the same time (15 min). Cells were rinsed twice in a potassium phosphate buffer solution and observed with an inverted Leica TCS SP5 microscope equipped with a 95% air–5% CO_2_-37°C thermostatic observation chamber and using a 40× objective. Green fluorescence from DCFH-DA and differential interference contrast were, respectively, excited at 488- and 561-nm wavelengths. Emission fluorescence levels and D.I.C. were detected using a photo-multiplicator between 498 and 561 nm, and a transmission photo-multiplicator, respectively. The two channels were recorded sequentially. Z series projections and fluorescence intensity measurements were performed using the ImageJ software (W. S. Rasband, ImageJ [U.S. National Institutes of Health, Bethesda, MD; http://rsb.info.nih.gov/ij, 1997–2009]).

### 4. Quantitative RT-PCR analysis of altered gene expression

The qPCR analysis was performed as described [21]. After exposure to each treatment condition, the total RNA was extracted from HEK293 cells by Total RNA Miniprep Kit (New England Biolabs). cDNA was prepared from 1 µg total RNA using SuperScript first-strand synthesis system (Thermo Fisher Scientific). Quantitative RT-PCR was performed using Luna qPCR master mix (New England Biolabs). Primers for oxidative stress-related genes GPX-1, GPX-3, SOD2, NOX-2, GSR, and CAT were as previously described [22]. In addition, we selected seven genes that had been previously shown to be regulated by both static and pulsed electromagnetic fields: KIAA1211, RPS16P5, TAS2R19 up-regulated after exposure to 10 Hz PEMF at 2 mT and KRT79, DDX50 and LINC01366 genes that were downregulated [20,21]. The GADPH gene was used as the reference gene. Quantitative RT-PCR was performed by Mastercycler RealPlex2 (Eppendorf). Three biological replicates were performed for each gene (N = 3). Data analysis to represent the relative expression level of genes of interest was performed as previously described [20,21]. Primers used for gene expression analysis are described in Table 1.

**Table 1.** Primer sequences

Gene		Primer sequence
***KIAA1211***	Forward	AGCTGGCTGTTAAGCCAAAA
	Reverse	CCTCCAGTTCTCGCCAGTAG
***RPS16P5***	Forward	TGCTAATGGCTGTGTGAAGC
	Reverse	GCCACAACAGGAAAAGGTGT
***TAS2R19***	Forward	GCAAACTGTGACCTCCTTCC
	Reverse	CGTGTCATCTGCCACAAAAC
***GADPH***	Forward Reverse	TGCACCACCAACTGCTTAGC GGCATGGACTGTGGTCATGAG
***KRT79***	Forward Reverse	GAGGAGAGCAGGATGTCTGG CGGTGCTATAGCCCACATTT
***DDX50***	Forward Reverse	GATGTCAGCTGTGCTTGGAA AGCCACTCCTCTGTCTGGAA
***LINC01366***	Forward Reverse	GCCCCTCTTTTCCTTCAATC TTGGCTGTGTTTCTGCAAAG
***UTS2B***	Forward Reverse	AAACGAGCTTGCTTTTGGAA GTCCAACCTGGCATTGTCTT
***CAT***	Forward Reverse	ACCCTCGTGGGTTTGCAGTGA CGAGCACGGTAGGGACAGTTCA
***GXP1***	Forward Reverse	TGGGCATCAGGAGAACGCCA GGGGTCGGTCATAAGCGCGG
***GXP3***	Forward Reverse	CTGACGGGCCAGTACATTGA TCCACCTGGTCGGACATACT
***GSR***	Forward Reverse	AGGAGCTGGAGAACGCTGGC CAATGGCCCAGAGCAGGCA
***SOD2***	Forward Reverse	GCAGCTGCACCACAGCAAGC CGTGCTCCCACACATCAATCCCC
***NOX2***	Forward Reverse	CAAGATGCGTGGAAACTACCTAAGAT TCCCTGCTCCCACTAACATCA

### 5. Statistical analysis

All data were analyzed using GraphPad Prism version 7.4.2 for Mac (GraphPad Software, La Jolla California, USA). Data were analyzed for normality with the Shapiro-Wilk test. The results will be expressed as mean ± standard error of the mean (SEM). The differences between treated and control conditions for each gene were compared using one-way ANOVA followed by Tukey's multiple comparison test. Comparisons were made of the Exposed (to a given static field condition) or Sham-exposed (to a canceled magnetic field condition) samples relative to the Control (−96 dBm background exposure) samples passaged at the same time from the same cell stock. Differences were considered statistically significant with a *p*-value <0.05 (*), <0.01 (**), <0.001 (***).

## Results

### Radiofrequency Field Exposure Triggers Increase in cellular ROS

The physiological consequences of non-thermal, man-made electromagnetic fields have been extensively studied, providing a clear trend that oxidative balance can be impacted by all of these signals. Nonetheless, reports on exposure to RF in the MHz – GHz (telecommunications) range continue to give inconsistent conclusions as to their effects on ROS signaling [23]. This problem largely arises from the diversity of experimental systems (cell types, exposure protocols, and readout assay protocols), which complicates efforts to determine whether a reported ”negative” result is in fact negative or instead due to suboptimal exposure or assay conditions.

In this study, we focus on the immediate effects of electromagnetic fields by assaying cellular response at a single frequency (1.8 GHz), while varying only the signal intensity. Furthermore, we have chosen rapid, clearly defined readouts for ROS signaling that occur after only a single brief exposure (production of cellular ROS and altered gene expression). This allows us to determine the initial response to RF as a function of RF signaling amplitudes.

This study was performed using HEK293 cells, a homogeneous immortalized model cell culture system that has been previously shown to respond to both static and pulsed electromagnetic fields [20,21]. Cells were maintained in culture dishes prior to exposure to 1.8 GHz signals produced by an antenna installed inside the incubator (methods). Our equipment consists of a single antenna installed in a CO_2_ incubator whose signal output is measured before and after each exposure cycle, in order to overcome possible artifacts due to equipment background or position effects. Both test and sham control cells were placed at identical positions in the incubator under the antenna, for identical times. Exposure was for one 15 min interval at the following amplitudes: Control (background): −96 dBm; High: −8.5 dBm; Medium: – 31 dBm; Low: – 55 dBm; Very Low: – 67 dBm. Repeated experiments were performed on different days and used different parent cell culture stocks to further reduce the possibility of artifact due to differences in cellular growth rates or conditions. During exposure, cell cultures were treated with DCFH-DC, a fluorescent dye which detects levels of •O_2_^−^, H_2_O_2_, HO^•^, and ONOO^−^. and was immediately subjected to confocal microscope analysis as described previously [21].

**Figure 2.**
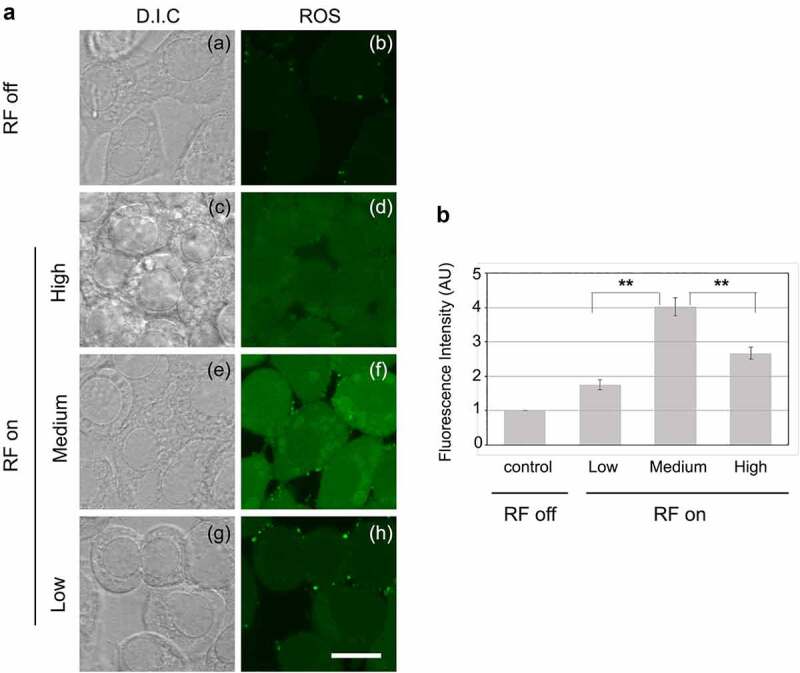
**Production and subcellular localization of ROS by HEK cells exposed to RF**. Living HEK cells were exposed or not to RF during 15 min and simultaneously treated with DCFH-DA [5-(and-6)-chloromethyl-2’,7’-dichlorofluorescein diacetate] and viewed by an inverted Leica TCS SP5 microscope. A: Images (a) to (h) show single confocal z section that cross the nucleus. Diffused fluorescent ROS staining can be seen in nucleus and cytoplasm at low, medium and high RF intensity. At medium and high RF intensity, additional vesicular and intense fluorescent ROS staining colocalizes perfectly with ER observed around the nucleus by differential interference contrast (D. I. C.). Scale bar 20 μm. B: Fluorescence intensities were measured cell by cell on z projected images, average values were normalized to the control condition (RF off) and shown as mean ± SD. Statistical analysis were performed as described (methods), n = 4. (** p < 0,01).

The results (Figure 2) show a marked increase in fluorescence in cell cultures after only 15 min of radiofrequency field exposure. Distribution of ROS was observed throughout the nucleus and the cytosol, with particularly high concentrations of vesicular structures of the golgi and endoplasmic reticulum surrounding the nucleus (Figure 2a). The distribution of ROS is similar to that caused by low-frequency (10 Hz) pulsed magnetic fields observed in prior studies [21]. Quantitation (Figure 2b) showed a nearly 3-fold increase in signal amplitude as compared to non-exposed controls.

The dose-dependence of the ROS accumulation was non-linear, showing the highest response at an intermediate amplitude of RF stimulation (‘Medium’ condition in Figure 2) and was decreased at both higher (High) and lower (Low and Very Low) amplitudes. The fact that ROS stimulation is maximal at an intermediate signal amplitude makes it unlikely to result from mechanical or thermal stresses, as these would be expected to increase linearly with signal amplitude. Taken together with the rapidity of its accumulation, induction of ROS by RF signals appears more consistent with a primary, possibly receptor-driven process.

### Radiofrequency Field modulates expression of Genes Implicated in oxidative stress

A number of well-characterized enzymes are known to degrade •O_2_^−^ and H_2_O_2_, whose transcription is rapidly up-regulated in response to an increase in intracellular ROS. These included catalase (CAT), superoxide dismutase (SOD), glutathione peroxidase (GPX), and glutathione-s-reductase (GSR) [22,23]. In addition, the NOX family (NADPH oxidases), which generate ROS, are also themselves transcriptionally regulated by ROS [24]. We accordingly evaluated members of each of these gene families for their response to RF exposure.

The exposure conditions were the same as those used for the ROS imaging experiments (above). HEK cell cultures were exposed to a single 15 min irradiation at the following signal amplitudes: Control (background): −96 dBm; High: −8.5 dBm; Medium: – 31 dBm; Low: – 55 dBm; Very Low: – 67 dBm. The cells were then returned to the incubator for an additional 3 hours to allow time for transcriptional regulation to occur, then harvested and analyzed by qPCR gene expression analysis.

**Figure 3.**
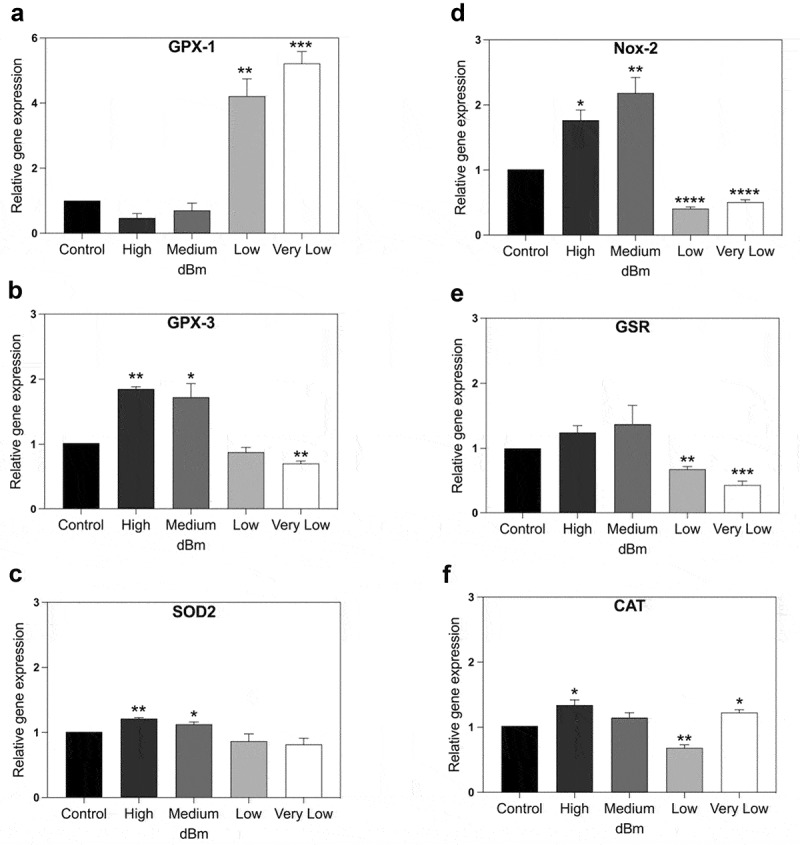
**qPCR gene expression analysis of genes implicated in control of cellular ROS. HEK** Cell cultures were exposed for 15 min to Control (background): −96 dBm; High: −8.5 dBm; Medium: – 31 dBm; Low: – 55 dBm; Very Low: – 67 dBm signal amplitudes. Gene expression was normalized to the Control (unexposed) conditions; statistical analysis was performed by ANOVA comparison of the exposed (High and Medium RF amplitude) to the Control (unexposed condition). Data are shown as mean ± SE of three independent experiments (N = 3). A – F. Expression of the indicated seven genes were compared at different amplitudes. Statistical analysis was performed as described (Methods). Significance level of the differences are as follows: *p-value < 0.1; ** *p*-value < 0.01; *** *p*-value < 0.01.

The results of qPCR analysis showed that RF triggered rapid change in expression of each of the tested ROS degrading enzymes GPX-1, GPX-3, SOD2, GSR, and CAT (Figure 3). This was to be expected since increased ROS has been shown to trigger an increase in concentration of ROS scavenging enzymes [22,24]. However, an unexpected feature of the response profile is that it varies greatly with respect to signal amplitude. GPX-1 showed a maximal expression peak only at the lowest (Very Low) signal amplitude, whereas GPX-3 and SOD2 responded maximally at the highest signal amplitudes; CAT in particular showed two peaks of expression, one at the highest (High) and one at the very lowest (Low) signal amplitude. Another unexpected result was that the expression of both GPX-3 and GSR significantly decreased at low signal strength (Very Low) as compared to the unexposed control condition even though higher signal amplitudes (and presumably higher levels of intracellular ROS) had no such effect (Figure 3).

In the case of Nox-2, enzyme whose activation results in synthesis of ROS, there was also a significant increase in gene expression after exposure to RF. This fits with the observation that increased ROS is produced in the cell after exposure to RF. The highest Nox-2 induction level occurs at Medium strength RF signal amplitude. This is the same exposure condition that triggered maximal cellular ROS (Figure 2), suggesting that Nox-2 may be somehow implicated in the production of ROS triggered by RF exposure. However, at lower signal amplitudes (Low and Very Low), the expression of Nox-2 was actually significantly downregulated. This response is in keeping with cellular defense mechanisms to return to low intracellular concentrations of ROS after the spike induced by RF exposure.

Taken together, all of these data show great complexity in the cellular response to RF signal, which can regulate the identical promoter in different directions depending on the signal amplitude. In this way, RF can potentially provide exquisite control over cellular redox homeostasis if the components mediating the response can be fully calibrated and characterized.

### Cellular response to RF is similar to response to applied magnetic fields

Previously, experiments had shown that exposure to as little as 15 min of a 10 Hz pulsed magnetic field (PEMF) resulted in an increase in intracellular ROS [21] similar to that induced by RF (Figure 2). We therefore investigated whether there might be some additional commonality between cellular responses to RF and to static magnetic fields. Prior experimental results from genome profiling had identified a number of genes specifically regulated by exposure to static or pulsed magnetic fields [20,21]. We therefore analyzed the expression characteristics of these known genes to determine whether they would also respond to radiofrequency fields.

**Figure 4.**
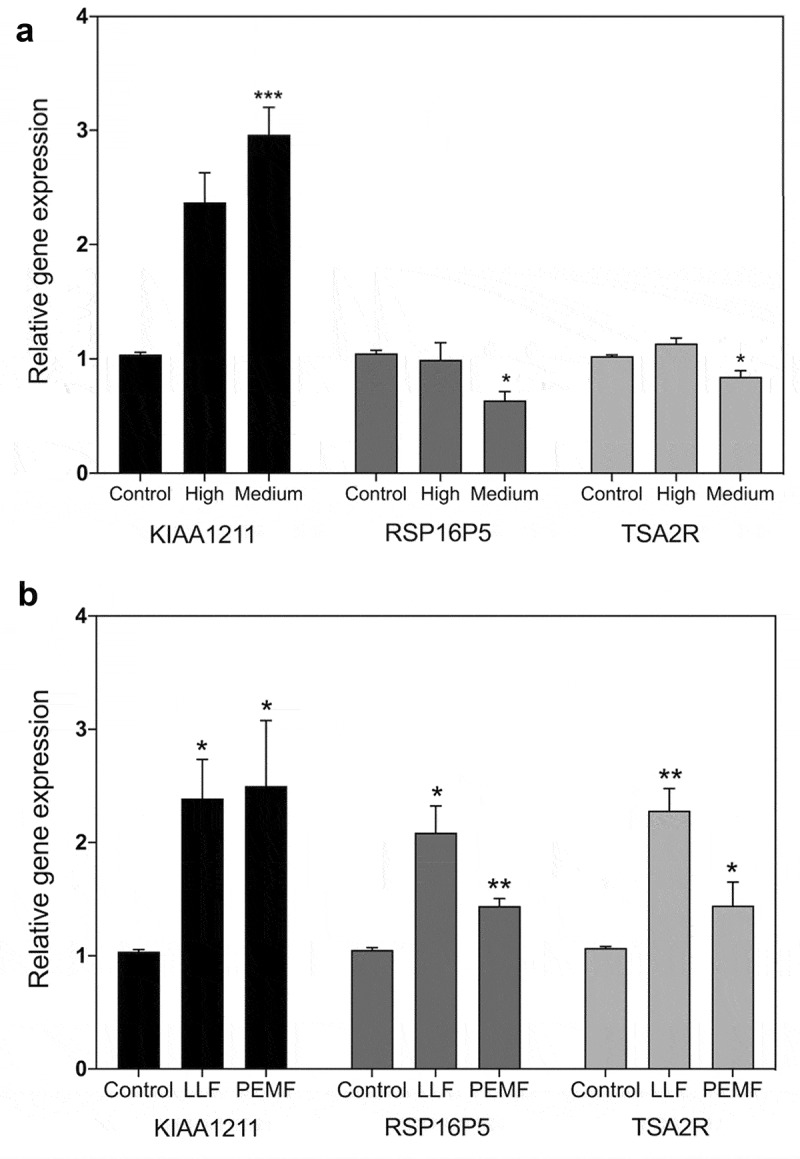
**qPCR gene expression analysis in HEK392 cells in response to 1.8 GHz or static magnetic field exposure. A**. Cell cultures were exposed to 15 min at the indicated amplitudes of 1.8 GHz RF field prior to harvest and RNA expression analysis (methods). Gene expression was normalized to the Control (unexposed) conditions; statistical analysis was performed by ANOVA comparison of the exposed (High and Medium RF amplitude) to the Control (unexposed condition). Data are shown as mean ± SE of six independent experiments (N = 6). **B**. Cell cultures were exposed to 15 minutes of either Low Level magnetic field (0.2µT) or to a Pulsed Electromagnetic Field prior to harvest and RNA expression analysis (methods). Gene expression was normalized to the Control condition; statistical analysis was performed by ANOVA comparison of the exposed (LLF and PEMF field) to the Control (unexposed condition). Data are shown as mean ± SE of three (LLF; N = 3) or six (PEMF; N = 6) independent experiments. The asterisks indicate significance level of the differences: *p-value < 0.1; ** *p*-value < 0.01; *** *p*-value < 0.01.

Figure 4 reports the results of qPCR expression analysis of three of these genes (KIAA1211, TSA2R, and RSP16P5) in response to both magnetic and radiofrequency fields. We tested their response to RF at signal amplitudes (High and Medium) at which a measurable increase in ROS was observable within the cells (Figure 2). One of these genes (KIAA1211) showed significant up-regulation (Figure 4a), whereas TSA2R and RSP16P5 genes showed no up-regulation and even a relative decline in activity in this amplitude range.

We next tested the same cultures used in RF field experiments for their response to static magnetic fields (Low Level and 10 Hz pulsed field). For the Low Level static magnetic field experiment, the cell cultures are briefly placed in a mu-metal funnel in the incubator such that the terrestrial magnetic field of 40 µT was decreased to less than 200 nT (see ref. 20 and Methods). We also tested the response to a 10 Hz Pulsed Electromagnetic Field; both these stimuli had been previously reported inducing intracellular ROS and stimulate expression of all three of the tested genes [20,21]. Consistent with prior studies, a significant increase in expression of the TSA2R and RSP16PS genes (Figure 3b) was observed in addition to KIAA1211, in response to static magnetic field exposure (LLF and PEMF; Figure 4b).

These results suggest that both magnetic fields and RF signals may have common cellular targets and work through fundamentally similar signaling mechanisms. However, since RF exposure induced an increase in expression in KIAA1211 but not in TSA2R and RSP16PS, this suggests a more nuanced response to radiofrequency signal amplitude.

### Biphasic Gene Expression Patterns occur as a function of RF signal amplitude

**Figure 5.**
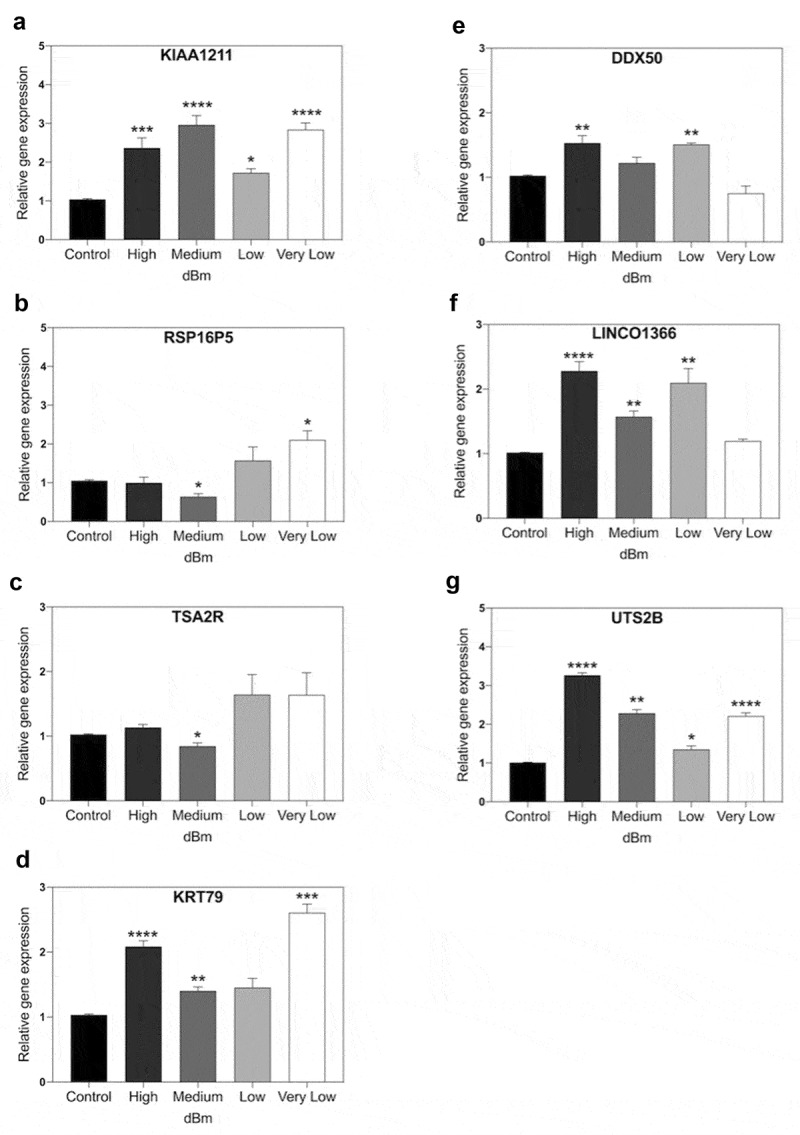
**qPCR gene expression analysis of RF signal amplitude dependence**. Cell cultures were exposed for 15 m to Control (background): −96 dBm; High: −8.5 dBm; Medium: – 31 dBm; Low: – 55 dBm; Very Low: – 67 dBm signal amplitudes. Gene expression was normalized to the Control (unexposed) conditions; statistical analysis was performed by ANOVA comparison of the exposed (High and Medium RF amplitude) to the Control (unexposed condition). Data are shown as mean ± SE of three independent experiments (N = 3). A – G. Expression of the indicated seven genes were compared at different amplitudes. Gene expression was normalized to the Control condition; statistical analysis was performed by ANOVA comparison of the different exposed (High, Medium, Low, or Very Low) to the Control (unexposed condition). The asterisks indicate significance level of the differences: *p-value < 0.1; ** *p*-value < 0.01; *** *p*-value < 0.01.

To provide a more comprehensive comparison, we performed a more extended RF dose response curve at 1.8 GHz, using also an additional magnetic field – responsive genes (from ref.21). The experimental procedure provides progressive changes in signal amplitudes from the background Control (at −96 dBm), through to Very Low (−67 dBm) and Low (−55 dBm), Medium (- 31 dBm), and on to the highest tested signal amplitude (High) at −8.5 dBm. The results from the analysis of seven representative magnetic field-responsive genes [21] at each of these four different signal amplitudes after 15 min exposure to 1.8 GHz are shown in Figure 5.

First, it was evident that, in contrast to the results of Figure 4a, changes in the expression of all three of the initially tested genes could be stimulated by RF fields. Even in the case of TSA2R and RSP16PS, which had not responded to High and Medium intensities, an increase in expression was triggered at the lower RF signal amplitudes (Low, Very Low) (Figure 5). Therefore, a 1.8 GHz signal could be used to increase expression of the very same genes that showed a response to static magnetic fields (20,21; – see also Figure 4b).

However, the kinetics of gene expression were more complex than a simple induction of expression. In fact, all of the tested genes gave indications of biphasic response patterns as a function of the signal amplitude. KIAA1211, for example, showed peak responsivity at Medium and Very Low amplitudes, but only a moderate induction at Low and Very High amplitudes (Figure 5). Similarly, the other six genes analyzed in this study all showed apparent decline at intermediate signal amplitudes, with maxima in expression near both extremes (Low and High amplitudes). What is particularly striking is how all of these genes without exception showed a peak of expression at lower signal amplitude, whereas two of them showed no appreciable response or even diminished expression, at the higher signal strength (RSP16P5, TSA2R).

These observations have profound implications for the interpretation of the current literature on RF effects. Not only is it evident that the exposure parameters are critical to the magnitude of a physiological outcome but it is also literally impossible to claim that ‘no physiological changes’ are triggered unless a full range of signal amplitudes are tested at each RF exposure condition.

## Discussion

In this study, our primary goal was to determine whether there could be direct physiological consequences to humans from exposure to radiofrequency fields generated by cell phones and Wi-Fi wireless devices and to evaluate a possible underlying response mechanism.

### 1.8 GHz RadioFrequency exposure results in rapid modulation of intracellular ROS

There have been many contradictory reports in the literature concerning physiological effects of RF exposure (see e.g. 23 and references therein). In large part, this is due to the complexity of wireless signals and the multiplicity of described physiological effects, involving vastly different experimental designs, controls, subjects, treatments, growth conditions (of cells), origins (of cells), and exhaustive range of biological assays (see e.g. 5–9). Nonetheless, modulation of ROS and ROS-related signaling pathways has been a recurring theme in this literature [10,11,25–31].

To achieve greater clarity on the question whether and how modulation of ROS in living cells is indeed a primary effect of exposure to RF, we assessed the immediate response to RF exposure in cell culture. We chose a simple model system (immortalized HEK cells) to assay for changes in ROS accumulation and ROS-regulated gene expression that occurred immediately after a single short, 15 min RF exposure period. We furthermore applied only a single, defined RF carrier frequency (1.8 GHz) to avoid effects due to signal complexity. The results showed both modulation of cellular ROS and rapid changes in ROS – regulated gene expression upon RF exposure. These data demonstrate that modulation of ROS is a direct and primary consequence of RF field exposure and that it is exquisitely sensitive to signal parameters.

Since ROS have so many effects on cellular responses (see e.g. 13–16), our results can provide an explanation for the many reports linking cellularfree radicals, oxidative stress, and ROS signaling to long term exposure to RF signals in a large variety of cellular types and organisms (see e.g. 10–12, 29–31).

### Telecommunications devices emit RF at sufficient amplitude to modulate intracellular ROS

A further goal of our study has been to determine the signal amplitude range over which radiofrequency fields can induce a physiological response.

To put RF signal amplitudes in perspective, a good Wi-Fi signal received at a tablet or cellular device is −50 to −70 dBm, while the router's antenna emits at +20-30 dBm. A cellular phone call can radiate up to +30-35 dBm at the start of the call and drop down to −20 dBm when the channel is optimized. The signal range in our current study (−8.5 to −76 dBm) lies well within these parameters. Changes in ROS concentrations via imaging experiments can be resolved down to −55 dBm (Figure 2), whereas rapid changes in the expression of ROS-regulated genes are observed throughout this whole range, including those for ROS scavenging and biosynthetic enzymes (Figures. 3, 5). We conclude that wireless signals in the GHz range, at amplitudes and exposure times for general public use, have definite intracellular consequences and cannot be considered physiologically inert.

Cell phones and routers have been classified as safe [3,4], and there is no conclusive evidence for pathology linked to these devices. However, given that even very low amplitude RF emissions trigger cellular production of ROS, it may nevertheless be possible that in isolated instances such devices could act synergistically with other forms of stress (e.g. pollution, UV radiation, pathology) to cause illness. Our data therefore suggest that there needs to be a reexamination of the question of safety in the context of other environmental or physiological factors that can also induce ROS and may act cumulatively with RF exposure.

### Positive and Negative Regulation of ROS Signaling Pathways occurs as a function of RF signal amplitude

An unexpected finding in our study has been the complexity of the biological response to RF signal amplitude. Instead of an expected linear curve in which the higher the RF amplitude, the greater (or less) the biological response, we instead find evidence of much more complex response characteristics.

For example, imaging experiments (Figure 2) show that maximal ROS accumulation occurs at an intermediate signal strength (Medium), but decreases at both higher and lower intensities (High and Low). ROS scavenging or synthetic enzyme gene expression also showed non-linear responses. In some cases both inhibition and induction maxima of identical genes were obtained at different RF signal amplitudes (Figure 3), whereas other of the analyzed genes actually showed a biphasic response characteristic in which gene expression was induced at lower and higher RF signal amplitudes yet was relatively unchanged at intermediate amplitudes (see e.g. CAT expression in Figure 3; all genes in Figure 5).

Such expression characteristics are referred to as ‘hormesis’ and have been identified as a feature of biological response to stimuli that are potentially toxic. These include heavy metals, drugs, hormones, and radiation exposure [32–35]. Biphasic gene expression characteristics have also recently been documented in response to static magnetic fields in plants [36], possibly also involving modulation of ROS through the cryptochrome photoreceptor. An explanation for the hormetic effects is that low concentrations of a toxin may specifically stimulate cellular defense and repair pathways, whereas high concentrations create more global stress, which triggers nonspecific response mechanisms. The hormetic effect is therefore consistent with the primary role of RF in stimulating ROS, which can be perceived by the cell as a toxin at high concentrations.

This complexity has profound implications for the interpretation of prior studies on RF effects in living systems. In fact, ‘absence’ of a given physiological response to RF may simply mean that the wrong signal frequency and/or amplitude has been used – to our knowledge, we are the first who have rigorously examined even the primary effects on ROS signaling of varying the signal amplitude over a short period of time. The fact that most prior studies have been conducted over longer periods of time, with vastly disparate signals at only one intensity, and investigating numerous downstream physiological effects in which ROS signaling can play multiple roles at many steps makes their interpretation virtually impossible.

### Mechanistic Considerations regarding RF effects in biological systems

Current theories suggest that RF triggers thermal or vibrational stresses in cells, which in turn indirectly lead to induction of ROS and other effects (10, 11, refs in 32). However, we detected no change in temperature in our cell cultures, and our data argue against the possibility of a simple mechanical effect due to the non-linear nature of the response. For instance, if increasing amplitude produces increasing vibration, then it is expected that the cellular response will simply increase as a function of amplitude. In fact, the strict amplitude dependence is more consistent with a biological receptor – mediated process. In particular, the biphasic response characteristics of gene expression, showing peaks of activity even at both low (Low) and higher (Medium) signal amplitude, do not appear consistent with such a simple thermal or mechanical mechanism (see e.g. Figs. 3–5).

An intriguing comparison is suggested by the many common features between cellular response to RF exposure and those of weak, near-earth strength magnetic fields [18–20,33,37,38]. This is because static magnetic fields have also been widely reported modulating cellular ROS [16–21], which are proposed to occur by a spin chemical mechanism whereby the magnetic fields can interact with the excited states of redox reactions to alter their ensuing quantum efficiency [39]. This so-called Radical Pair mechanism is moreover shown to be modulable by Radiofrequency fields in the MHz range. However, while common hyperfine interactions, such as those formed by flavoproteins, are of the order of 1 mT ~ 28 MHz, no energy-level differences are expected beyond ~100 MHz and, thus, no sensitivity of radical pairs to 1.8 GHz radiation can result. Therefore, the radical pairing mechanism as it is currently understood cannot account for effects in the GHz range, since a completely novel chemical signaling response pathway would be required to explain resonance effects in this frequency range. Nonetheless, the ultimate biological effects appear to be similar given that ROS is stimulated both by static magnetic fields and by GHz radiation.

Irrespective of the precise mechanism of reception, it is clear that the response of RF in the 1.8 GHz range is extraordinarily finely tuned and can independently regulate different genes in different ways. In particular, it is possible to both positively and negatively regulate the same gene simply as a function of signal amplitude, giving the possibility to non-invasively, sequentially activate and deactivate cellular pathways of medical interest on any desired timescale. Although in this study, we have only used a single frequency to provide proof-of-principle data, it is likely that specific control of many genes could be elicited by different frequencies, waveforms, and signal intensities. In this case, our findings could lead to a powerful new technology with unprecedented applications in medicine and in biotechnology.

## Conclusion

In conclusion, we show that exposure to RF within the amplitude range emitted by household telecommunication devices can have a direct and immediate physiological effect on cellular ROS biosynthesis and signaling. This response is dependent in complex ways on RF signal amplitude, consistent with a biological receptor mediated process. The response to RF further shares common features with modulation of ROS by static magnetic fields. Since ROS regulates oxidative stress and cellular signaling and response pathways, our results provide a possible mechanistic explanation for the many different reported physiological effects of RF in the literature.
